# Genetic deletions and high diversity of *Plasmodium falciparum* histidine-rich proteins 2 and 3 genes in parasite populations in Ghana

**DOI:** 10.3389/fepid.2022.1011938

**Published:** 2022-10-14

**Authors:** Nancy Odurowah Duah-Quashie, Philip Opoku-Agyeman, Selassie Bruku, Tryphena Adams, Kwesi Zandoh Tandoh, Nana Aba Ennuson, Sena Adzoa Matrevi, Benjamin Abuaku, Neils Ben Quashie, Chaselynn Watters, David Wolfe, Hugo Miranda Quijada, Terrel Sanders

**Affiliations:** ^1^Department of Epidemiology, Noguchi Memorial Institute for Medical Research, College of Health Sciences, University of Ghana, Accra, Ghana; ^2^West African Center for Cell Biology of Infectious Pathogens, Department of Biochemistry, Cell and Molecular Biology, College of Basic and Applied Sciences, University of Ghana, Accra, Ghana; ^3^Centre for Tropical Clinical Pharmacology and Therapeutics, University of Ghana Medical School, College of Health Sciences, University of Ghana, Accra, Ghana; ^4^US Naval Medical Research Unit No. 3, Ghana Detachment, Accra, Ghana

**Keywords:** malaria, rapid diagnostic tests (RDTs), *Plasmodium falciparum* histidine rich protein-2 (PfHRP-2), *Plasmodium falciparum* histidine rich protein-3 gene (pfhrp3), amino acid repeat types

## Abstract

Rapid diagnostic tests (RDTs) are used to diagnose malaria in Ghana and other malaria endemic countries. *Plasmodium falciparum* histidine-rich protein 2 (PFHRP2*)* based RDTs are widely used, however the occurrence of deletions of the *pfhrp2* gene in some parasites have resulted in false negative test results. Monoclonal antibodies of PFHRP2 cross reacts with PFHRP3 because they share structural similarities and this complements the detection of the parasites by RDT. These two genes were investigated in Ghanaian *P. falciparum* parasite population to detect deletions and the polymorphisms in exon 2 of the *pfhrp2* and *pfhrp3* genes. Parasite isolates (2,540) from children ≤ 12 years with uncomplicated malaria from 2015 to 2020 transmission seasons were used. Both genes were amplified using nested PCR and negative results indicated the presence of the deletion of genes. Amplified genes were sequenced for the detection of the amino acid repeats. Deletions were observed in 30.7% (780/2,540) and 17.2% (438/2,540) of the samples for *pfhrp2* and *pfhrp3* respectively with increasing trends over the three time periods (χ2 −10.305, *p* = 0.001). A total of 1,632 amplicons were sequenced for each gene, analysis was done on 1,124 and 1,307 good quality sequences for *pfhrp2* and *pfhrp3* respectively. *Pfhrp2* repeat polymorphisms were dominantly of types 2 (AHHAHHAAD) and 7 (AHHAAD) with large numbers of variants. A novel variant of type 14 (AHHANHATD) was seen for *pfhrp2*. For the *pfhrp3* repeat types, 16 (AHHAAN), 17 (AHHDG) and 18 (AHHDD) were the dominant types observed. Variants of type 16 (AHHAAH) and (AHHASH) were also dominant. Repeat types 1, 2, 3, 4, 5, 6, 7, 8, 11, 13, 15, 16, and 19 were observed be shared by both genes. The haplotype diversity of both genes ranged between 0.872 and 1 indicating high diversity of the polymorphisms in the isolates. The implication of the findings of the frequencies of the *pfhrp2* and *pfhrp3* deletions as well as the variants of the main epitopes of the monoclonal antibodies for the RDT (types 2 and 7) in our isolates is an indication of decreased sensitivity of the RDTs in diagnosing malaria infections in Ghana.

## Introduction

The devastating effect of malaria on vulnerable populations in disease endemic countries is still overwhelming. An estimated 241 million malaria cases and 6,27,000 malaria deaths worldwide was recorded in 2020 which portray an increment of about 14 million cases and 69,000 deaths from estimates for 2019 (1). For the World Health Organization (WHO) African Malaria Region, between 2019 and 2020, estimated malaria cases increased from 213 million to 228 million, and deaths from 5,34,000 to 6,02,000 (of which 80% of all deaths were among children ≤ 5 years) (2). The T3 initiative for ‘Test. Treat. Track.' by the WHO Global Malaria Programme was implemented to help endemic countries reduce disease burden by diagnostic testing, antimalarial treatment, and surveillance systems strengthening (3). Rapid and correct diagnosis of malaria is therefore crucial before any form of therapy could be administered for indigenes living in these areas, tourists visiting these areas and military troops on assignment to these endemic regions.

The gold standard malaria diagnosis tool is microscopy which is quite laborious and not time efficient for rapid results (4). The introduction of rapid diagnostic tests (RDTs) for malaria diagnosis was important due to the ease in its use, does not require expert rigorous training and gives quick results in 15 min (4). These tests detect malaria parasite proteins, soluble PFHRP2 and PFHRP3 in infected host's blood through immunochromatographic lateral flow methods and are used globally as a field-deployable diagnostic tool for both symptomatic and asymptomatic infections (5). RDT use from 2010 to 2020 is estimated to be 3.1 billion of which about 81% of these were procured by sub-Saharan African countries (2, 5). So far, the most sensitive and most used RDT for *P. falciparum* malaria is the Histidine rich protein 2 (PFHRP2)-based tests (5). The PFHRP2 is a highly expressed and abundant protein secreted by the parasite during the blood stage because of the haem-binding site to mediate the formation of haemozoin. The abundance of PFHRP2 in blood during infection, its specificity as to being expressed only by *P. falciparum* as well as its thermal stability makes the protein an excellent target for diagnosis (6–9). The sensitivity of the test is increased by the presence of the repetitive epitopes of the protein which enhances detection by antibodies (6). A new type of ultra-sensitive RDTs with a minimum detection limit of <100 parasites/μl is based on the PFHRP2 (10). Other RDTs identifying other parasite proteins, such as lactase dehydrogenase (pLDH) and aldolase are available, however, these are comparatively less sensitive (11).

PFHRP2-based RDTs are able to identify a structurally similar protein PFHRP3 in the blood of a malaria infected person. These two soluble proteins, PFHRP2 and PFHRP3, share multiple epitopes, have two exons separated by an intron and are encoded by *pfhrp2* and *pfhrp3* genes located on chromosome 8 and 13 respectively in the genome of *P. falciparum* (12, 13). The first exon encodes a signal peptide whilst the second exon, the main coding region, encodes specific histidine-alanine rich repeats (12, 14, 15). The first report of the *pfhrp2* and *pfhrp3* deletions was made by Gamboa and her group in Peru (16) and to date many reports on the co-deletions and amino acid repeats variations have been made from malaria endemic countries worldwide which threatens disease eradication efforts (13, 17–19). The accuracy of PFHRP2-based RDTs is greatly compromised by the deletion of the *pfhrp2* gene with the consequent false-negatives results (20). Additionally, the sensitivity of these RDTs has greatly been affected by parasite density, the lack of PFHRP2 expression and the genetic variability in the amino acid repeats encoded by *pfhrp2* and *pfhrp3* (21).

In Ghana, a study conducted in the southern part of the country gave the first report of *pfhrp2* deletions (22). The deletions were observed in 33 and 36% of microscopically confirmed and PCR-confirmed RDT positive samples respectively. The group recently reported 12.9% and 39.5% deletions in the partial coding regions (exon 1–2) and main coding region (exon 2) of *pfhrp2* respectively with 5.2 and 40.5% of these deletions attributed to *pfhrp3* (23). Another study in Ghana observed no *pfhrp2* deletions in a study conducted on 50 school children in the Volta region of the country but observed 12 amino acid repeat types, of which two were novel and therefore the first report of genetic variation of *pfhrp2* in Ghana (24). It is needful for continued surveillance to determine the extent of the polymorphisms in the two genes as part of the country level malaria elimination efforts with the long-term goal of safeguarding accurate malaria diagnosis and prompt treatment. This study described polymorphisms in *pfhrp2* and *pfhrp3* in malaria parasite population in Ghana from 2015 to 2020 and determined their genetic evolution over time and possible selection pressures as well as their implications on the use of RDTs in Ghana.

## Materials and methods

### Study sites

**Figure 1:**
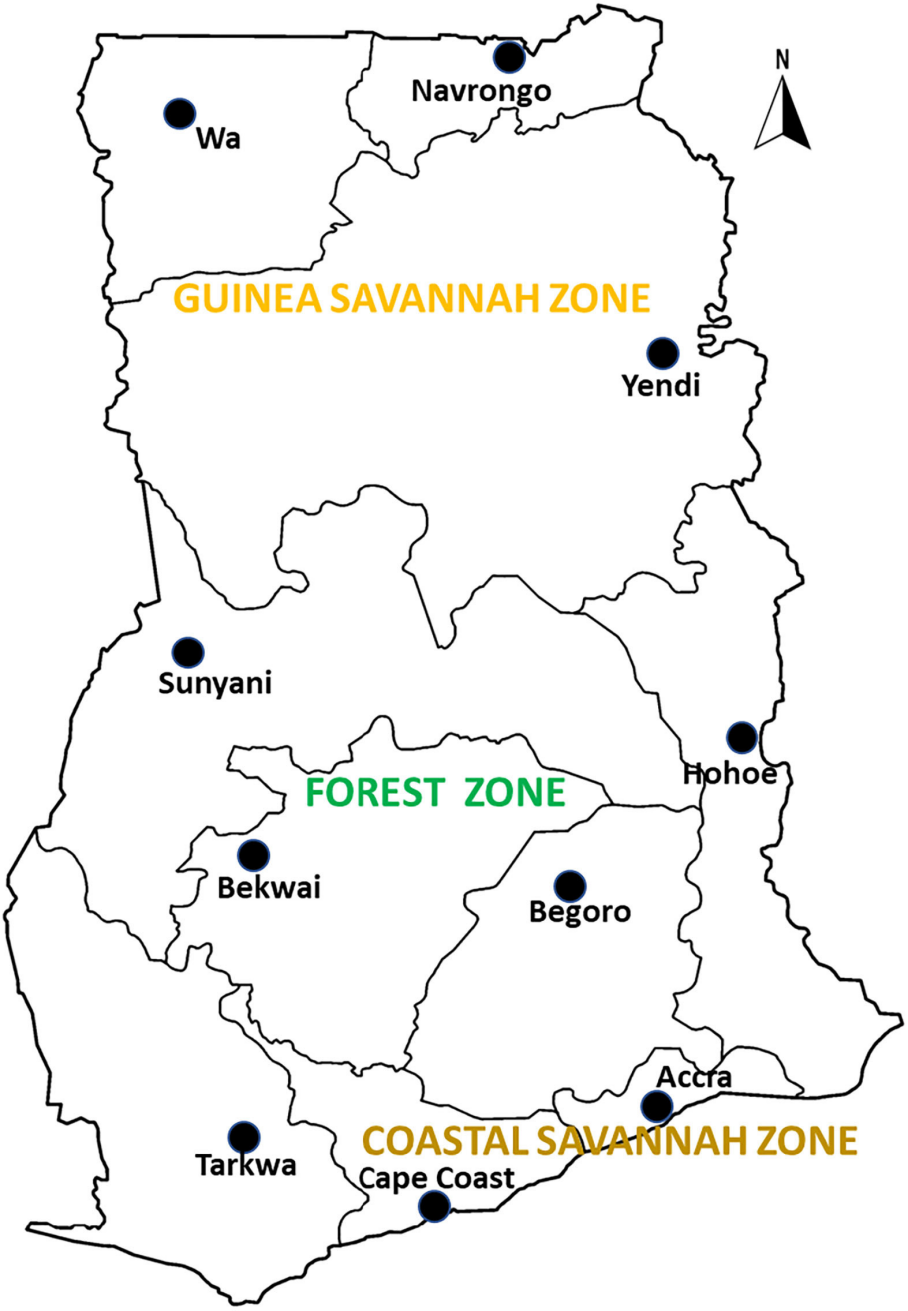
A map of Ghana showing the study sites in the ecological zones. The study sites are sentinel sites for monitoring antimalarial drug efficacy/resistance in Ghana in the 10 regions of the country. These sites have hospitals and health centers for malaria Treatment Efficacy Studies, and were set up by a collaboration between the National Malaria Control Program and NMIMR.

Archived samples from therapeutic efficacy studies (TES) conducted in 10 sentinel sites representing three different ecological zones of Ghana namely coastal, forest and savannah were used for the study. The sentinel sites located in the forest zone with perennial malaria transmission are Begoro (6.3916°N, 0.3795°W), Bekwai (6.4532°N, 1.5838°W), Hohoe (7.1519°N, 0.4738°E), Sunyani (7.3349°N, 2.3123°W) and Tarkwa (5.3018°N, 1.9930°W); guinea savannah zone with seasonal malaria transmission are Navrongo (10.8940°N, 1.0921°W), Wa (10.0601°N, 2.5099°W) and Yendi (9.4450°N, 0.0093°W); coastal savannah with perennial malaria transmission are Accra (5.6037°N, 0.1870°W) and Cape-Coast (5.1315°N, 1.2795°W) (Figure 1).

Malaria transmission in the guinea savannah zone is seasonal variation and estimated annual entomological inoculation rate (EIR) of up to 1,132 infective bites per person per year (25). For the forest zone transmission is intense and perennial with estimated annual EIR of up to 866 infective bites per person per year (25). For the coastal zone, malaria transmission is perennial but not intense with estimated annual EIR of fewer than 50 infective bites per person per year (25). According to the Ghana Malaria Indicator Survey (MIS), in 2019, the prevalence of the disease ranged between 10 and 19% for the guinea savannah zone, 10–27% for the forest zone and 2–18% for the coastal savannah zone (26).

### Study population and samples

Archived filter paper blood blots, collected from children aged 12 years and below reporting at the health centers at the sentinel sites with uncomplicated malaria from 2015 to 2020 malaria transmission seasons were used for the study. The blood samples were collected onto a Whatman^TM^ filter paper (Little Chalfont, UK), and the dried bloodspots (DBS) stored in plastic bags containing silica gels, and kept at room temperature until use. Parents/guardians of the children gave written informed consent for their participation in the TES. The consent also covered the future use of the archived samples for further molecular analysis.

### Parasite DNA extraction and molecular analysis

Genomic DNA was extracted from the DBS using QIAamp DNA Mini Kit (QIAGEN, Germany) following manufacturer's instructions and kept at −20°C until use. Nested PCR was used to amplify the exon 2 region of both the *pfhrp2* and *pfhrp3* genes according to previously published protocol with minor modifications (13). The parasite strains 3D7 (*pfhrp2*+ and *pfhrp3*+), DD2 (*pfhrp2-* and *pfhrp3*+) and HB3 (*pfhrp2*+ and *pfhrp3*–), were used as controls for the study. For confirmation of gene deletions, negatively amplified samples were re run thrice to confirm deletions. Further analysis were done using *pfk13* (27) and *pfmsp2* (28) gene amplification to confirm parasite DNA presence in those samples. Successfully amplified samples were Sanger sequenced by Macrogen, Europe (Netherlands).

### Data analysis

The chi-squared test for trend analysis was used to determine the trend of the prevalence of the deletions over the timepoints with StatCalc in Epi Info^TM^ 7.2.5.0 (Centers for Disease Control and Prevention, Atlanta, USA). Sequences obtained were run in the Basic Local Alignment Tool (www.ncbi.com) to check for authenticity of the sequence data. Parasite DNA sequences of *pf* hrp2 and *pf* hrp3 were aligned to 3D7 wildtype reference sequences with accession numbers PF3D7_0831800 and PF3D7_1372200 respectively. Detection and manual counts of the amino acid repeat types [as described by Baker et al. (12)] and sub-variants [as described by Nderu et al. (29)] were done by translating nucleotides sequences into amino acids using the CLC Main Workbench 22.0.1 software (Qiagen, Aarhus, Germany) and the Benchling website (San Francisco, CA, USA). For automated counts of the amino acid variants for each sample, base-calling, alignment, and deconvolution of sanger chromatogram trace files were also done using the command-line version of the application Tracy (30). The output binary variant call format (bcf) files for each sample were converted to human-readable variant call format (vcf) files using custom bash scripts. Low-quality nucleotide variants (Phred score <40) were filtered out from the vcf file. After this, the fasta file for each sample was generated from the output vcf files using custom bash scripts. The protein translation was done using the EMBOSS-transeq function in customized bash scripts (https://www.bioinformatics.nl/cgi-bin/emboss/transeq). Finally, exploratory data analysis, graphical summaries and statistical analysis were done using custom R scripts in R version 4.0. Fasta files were fed into DnaSP version 6.10.01 to determine haplotype diversity (31). Chi squared test was used to test for association between the predictor variables, ecological zones and the response variable as predicted RDT sensitivity for the Baker's model analysis (14).

## Results

### Prevalence of *P. falciparum* isolates with *Pfhrp2* and *Pfhrp3* deletions

**Figure 2:**
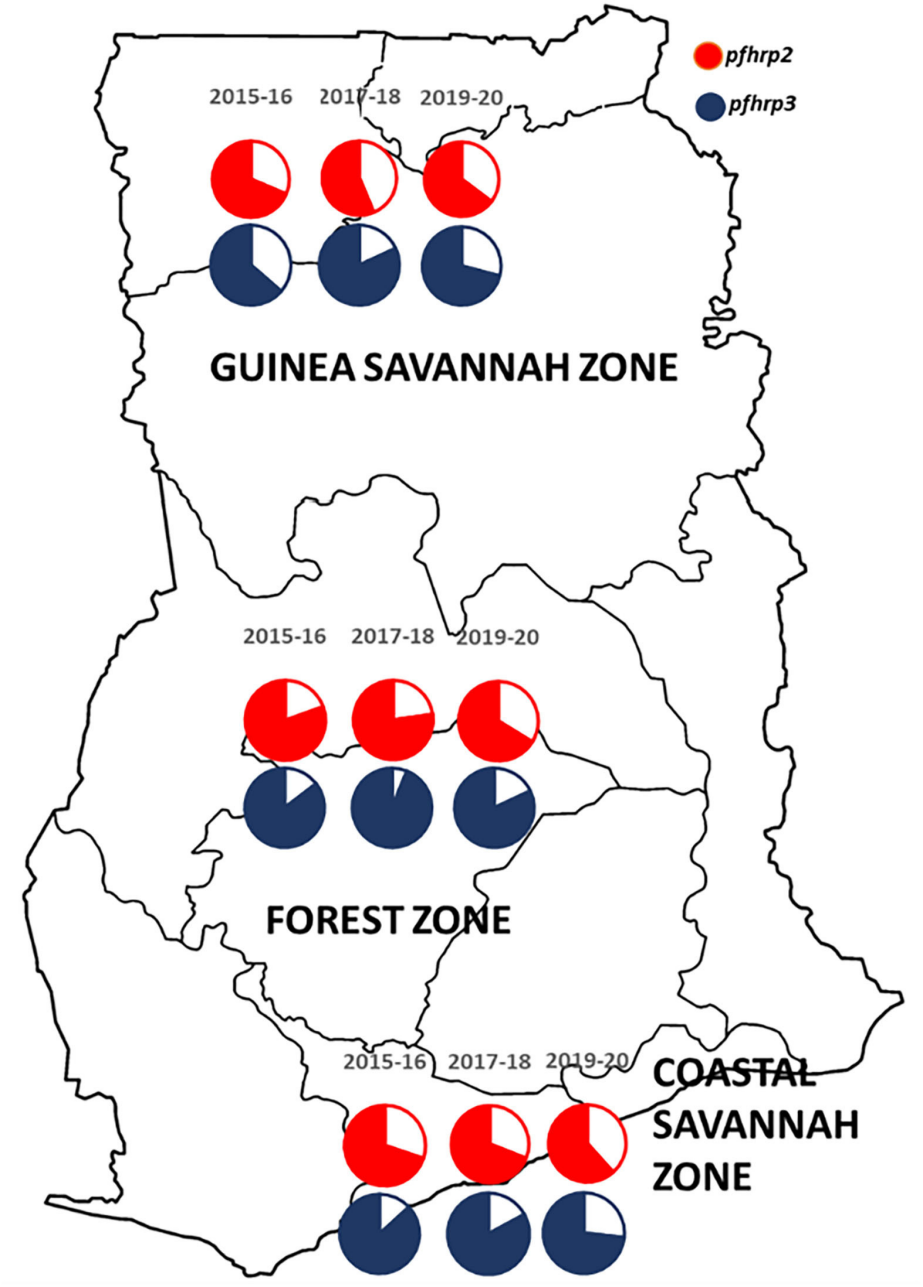
A map of Ghana showing the distribution of the prevalence of deletions in the *pfhrp2* and *pfhrp3* in the three ecological zones over the three time points. The white/plain segments of the pie charts are the percentage deletions for *pfhrp2* (red) and *pfhrp3* (blue-black).

A total of 2,540 samples were successfully analyzed for *pfhrp2* and *pfhrp3* gene amplifications. Out of these, 69.3% (1,760/2,540) and 82.8% (2,102/2,540) samples were positive for *pfhrp2* and *pfhrp3* respectively. Repeated PCRs were done for samples with no positive results to ascertain the presence of the deletions. In addition, the genes, *pfk13* and *pfmsp2*, were amplified to ascertain presence of parasite DNA in the negative PCRs for the *pfhrp2* and *pfhrp3*. The deletions were therefore observed as 30.7% (780/2,540) and 17.2% (438/2,540) respectively for *pfhrp2* and *pfhrp3*. The distribution of samples by time points and the prevalence of deletions are shown in Table 1. The chi-square for trend analysis showed a significant increasing trend for the prevalence of the deletion in *pfhrp2* (χ2 −10.305, *p* = 0.001) but not for *pfhrp3* (χ2 −0.557, *p* = 0.455) over the three time points. The samples numbers as per ecological zone per year is as follows: Guinea savannah zone (GS) −105, 270, and 343 for the time points 2015–16, 2017–18, and 2019–20 respectively; Forest zone (FS) −267, 603, and 518 respectively for time points 2015–16, 2017–18, and 2019–20; Coastal savannah zone (CS) – 159, 129, and 146 for timepoints 2015–16, 2017–18, and 2019–20 respectively. The prevalence of deletions in *pfhrp2* and *pfhrp3* in the three ecological zones over the three time points are shown in Figure 2.

**Table 1:** Number of samples per year and the prevalence of deletions of *pfhrp2* and *pfhrp3* genes.

		**Deletions**	
**Time points**	**No. of samples**	***pfhrp2*/% (no.)**	***pfhrp3/*% (no.)**
2015–2016	531	25.0 (133)	18.5 (98)
2017–2018	1,002	29.4 (295)	11.0 (108)
2019–2020	1,007	35.0 (352)	23.0 (232)
Total	2,540	30.7 (780)	17.2 (438)

The proportion of isolates with the deletions of the two genes varied from year to year for both genes with statistically significant increasing trends for pfhrp2 (χ2 −10.305, p = 0.001).

### Amino acid repeat polymorphisms in *the pfhrp2* and *pfhrp3* genes

A total of 1,632 amplicons were sequenced, out of which 1,124 and 1,307 good sequences were used for the genetic analysis respectively for *pfhrp2* and *pfhrp3*. Seventeen (17) of the 24 amino acid repeat types [as characterized by Baker et al. (12) and Nderu et al. (29)] were observed in the parasite isolates for *pfhrp2* and *pfhrp3* as shown Table 2. Amino acid repeat types observed were types 1–21 in Ghanaian isolates for both genes. The types 2 and 7 of the *pfhrp2* were dominant types observed with consequent high numbers of variants of 97 and 38 respectively. A similar observation was made for *pfhrp3*, types 16 and 17 dominated with high numbers of variants, 38 and 20 respectively. The number of variants for each amino acid type and the frequencies of the predominant variants are shown in Table 3 for the two genes. The numerous variants of the amino acid types including both known and novel variants are documented in Supplementary Tables 1, 2. Of the known 24 amino acid repeat types, 13 repeat types, 1, 2, 3, 4, 5, 6, 7, 8, 11, 13, 15, 16, and 19 were observed to be shared by both genes. However, the types 10, 12, 14, and 21 were uniquely found in the *pfhrp2* whilst 9, 17, 18, and 20 were exclusive for *pfhrp3*.

**Table 2:** Amino acid types of *pfhrp2 and pfhrp3* and the range of observed repeats in Ghanaian isolates.

		**Range of occurrence of repeats in isolates**	
**Amino acid repeat type**	**Repeat sequence**	***pfhrp2***	***pfhrp3***
1	AHHAHHVAD	0–4	0–4
2	AHHAHHAAD	0–19	0–1
3	AHHAHHAAY	0–3	0–1
4	AHH	0–10	0–4
5	AHHAHHASD	0–3	0–1
6	AHHATD	0–10	0–1
7	AHHAAD	0–12	0–11
8	AHHAAY	0–3	0–2
9	AAY	-	0–2
10	AHHAAAHHATD	0–4	-
11	AHN	0–1	0–1
12	AHHAAAHHEAATH	0–1	-
13	AHHASD	0–5	0–1
14	AHHAHHATD	0–2	-
15	AHHAHHAAN	0–2	0–2
16	AHHAAN	0–1	0–16
17	AHHDG	-	0–11
18	AHHDD	-	0–5
19	AHHAA	0–1	0–1
20	SHHDD	-	0–1
21	AHHAHHATY	0–1	-

The variability of repeat sequences for reported amino acid types for pfhrp2 and pfhrp3 ranged between 0 to 19 repeat sequences depending on the amino acid type. Type 2 had the highest repeat sequence occurrence for pfhrp2 and types 7 had the highest for pfhrp3.

**Table 3:** Variants of amino acid repeat types observed in *pfhrp2* and *pfhrp3*.

**Amino acid repeat types**	***phrp2***		***phrp3***	
	**Observed no. of variants per type**	**Prominent variants (no. of samples)**	**Observed no. of variants per type**	**Prominent variants (no. of samples)**
1 AHHAHHVAD	25	AHHAHHVAH (15)	19	npv
2 AHHAHHAAD	97	AHHAHHAAH (110) AHHAHHADD (25) AHHAHHAHD (55) AHHAHHAHH (214) AHHAHHAPD (276) AHHAHHAPH (402) AHHAPHAHH (86) AHHAPHAPH (26) APHAHHAPH (37) APHAHHAHH (13) APHAPHAPH (10)	-	-
3 AHHAHHAAY	8	AHHAHHAPY (57)	1	npv
4 AHH	3	APH (11)	6	npv
5 AHHAHHASD	6	AHHAHHASH (51)	-	-
6 AHHATD	2	AHHATH (22)	-	-
7 AHHAAD	37	AHHAAH (44) AHHADD (26) AHHADH (12) AHHAHD (56) AHHAPD (121) AHHAPH (246) APHAHH (44) APHAPH (10)	4	npv
8 AHHAAY	8	AHHAPY (22)	8	npv
10 AHHAAAHHATD	36	npv	-	-
12 AHHAAAHHEAATH	18	npv	-	-
13 AHHASD	1	npv	-	-
14 AHHAHHATD	3	npv	-	-
15 AHHAHHAAN	1	npv	10	npv
16 AHHAAN	-	-	38	AHHAAH (208) AHHAPH (149) AHHATN (38)
17 AHHDG	-	-	20	AHHDE (63) AHHNA (244) PHHDG (33) SHHDG (22)
18 AHHDD	-	-	13	PHHDD (19)
20 SHHDD	-	-	6	SHDDH (14) SHHDH (83)

There was enormous variability in the amino acid repeat types 2 and 7 for pfhrp2. Some of the variants especially for type 2 were observed in 402 isolates sequenced.

Amino acid repeat type not observed.

npv, no prominent variants observed.

### Amino acid size variation in exon 2 of *Pfhrp2* and *Pfhrp3*

**Figure 3:**
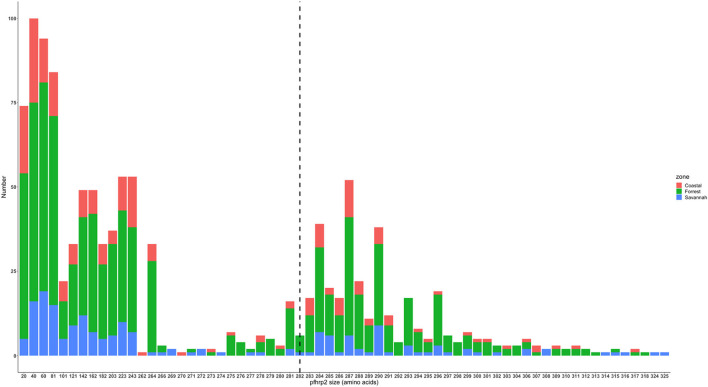
*Pfhrp2* exon 2 amino acid (aa) size distribution in isolates from the three ecological zones. A total of 1,124 sequences analyzed depicts the extent of insertions/deletions in variants in size of *pfhrp2* exon 2. The highest frequency (mode) was 40 aa found in 100 samples analyzed. The 3D7 reference amino acid size of *pfhrp2* exon 2 is 282 amino acids and is demarcated by the black dashed line. The bar plots are stacked by the three transmission zones.

**Figure 4:**
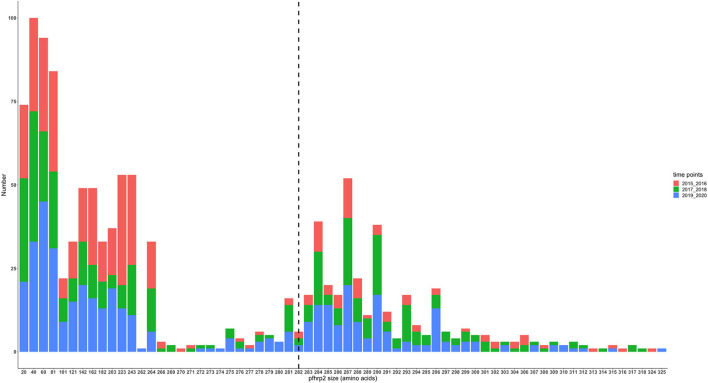
*Pfhrp2* exon 2 amino acid (aa) size distribution in isolates from the three time points. The 3D7 reference amino acid size of *pfhrp2* exon 2 is 282 amino acids and is demarcated by the black dashed line. The bar plots are stacked by the time periods of sample collection.

**Figure 5:**
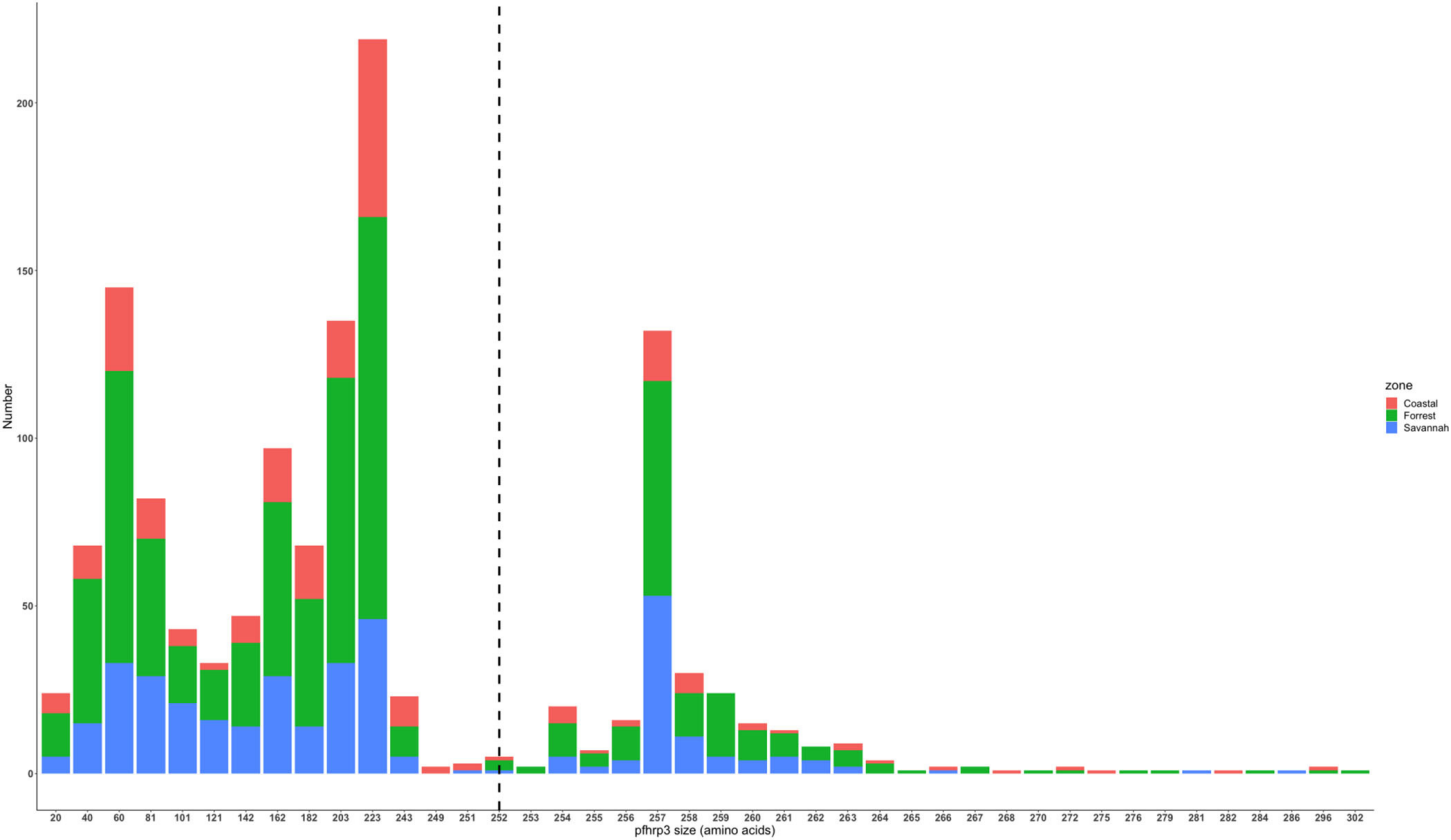
*Pfhrp3* exon 2 amino acid (aa) size distribution in isolates from the three ecological zones. A total of 1,293 sequences analyzed depicts the extent of insertions/deletions in variants in size of *pfhrp3* exon 2. The highest frequency (mode) was 223 aa found in 219 out of the 1,293 samples analyzed. The reference amino acid size of *pfhrp3* exon 2 is 252 amino acids and is demarcated by the black dashed line. The bar plots are stacked by the three ecological zones.

**Figure 6:**
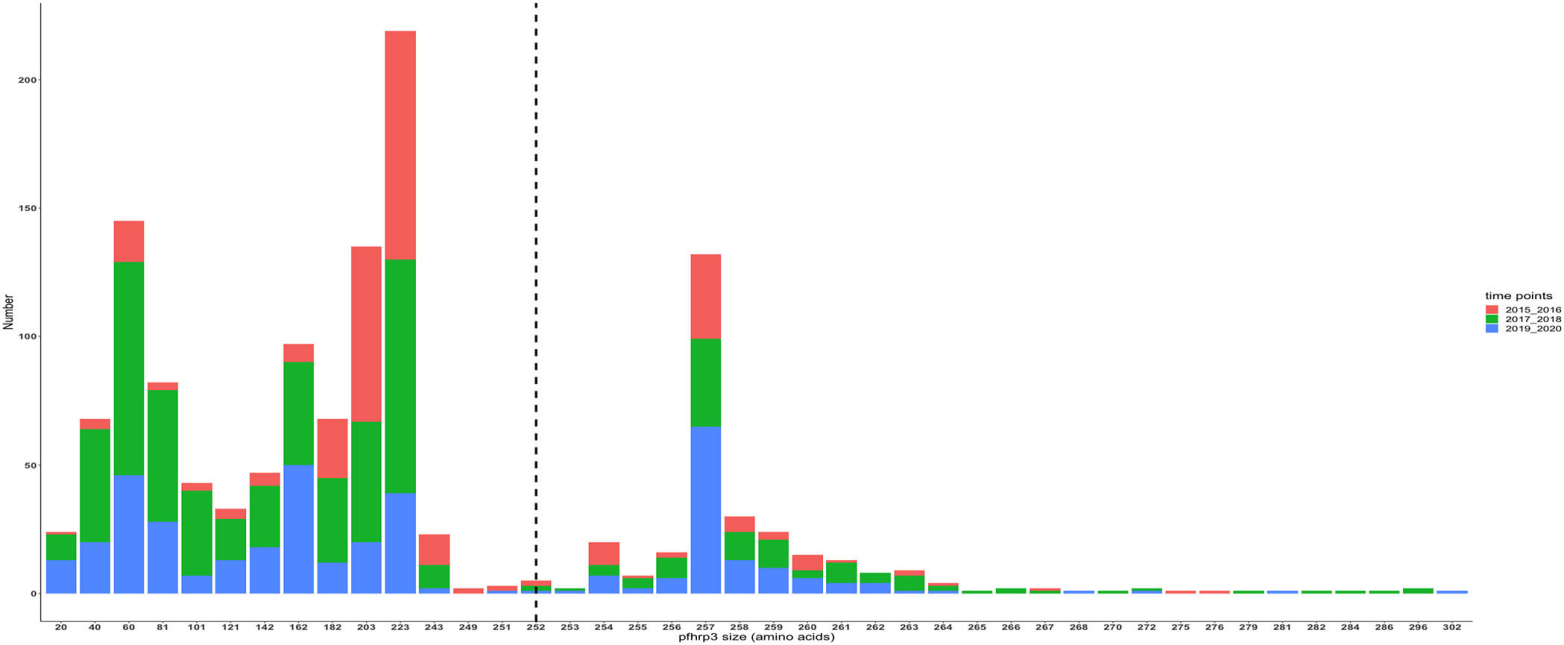
*Pfhrp3* exon 2 amino acid (aa) size distribution in isolates for the three time points. The reference amino acid size of *pfhrp3* exon 2 is 252 amino acids and is demarcated by the black dashed line. The bar plots are stacked by the time periods of sample collection.

**Figure 7:**
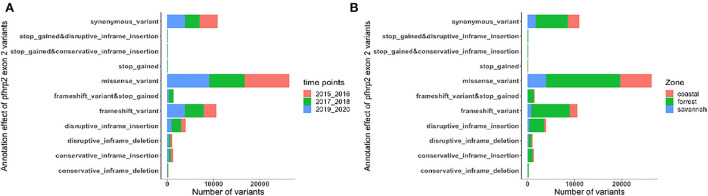
Distribution of annotation effect of all genetic variants observed in *pfhrp2*. The annotation effects from 1,12,524 genetic variants from all the *pfhrp2* exon 2 sequences analyzed. Bar charts are stacked by **(A)** sampling time points and **(B)** ecological zones.

**Figure 8:**
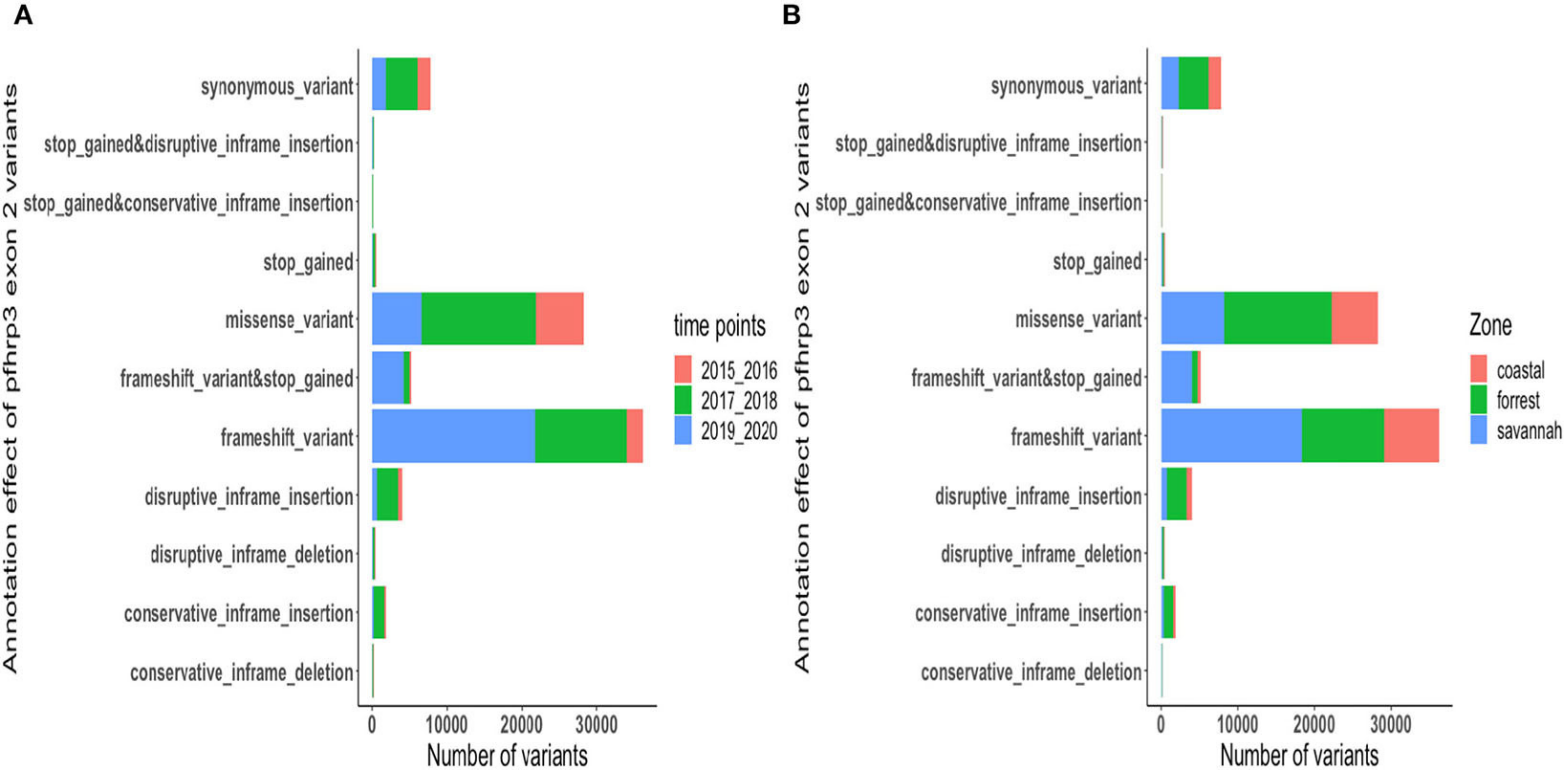
Distribution of annotation effect of all genetic variants observed in *pfhrp3*. The annotation effect of 88,926 genetic variants from all the *pfhrp3* exon 2 sequences analyzed. Bar charts are stacked by **(A)** sampling time points and **(B)** ecological zones.

The translated nucleotide sequences varied in size from 20 to 325 amino acids for *pfhrp2* and 20 to 302 for *pfhrp3* exon 2 (Figures 3–6). The analysis showed that most of these variations in amino acid repeats were caused by missense, frameshift and insertions/deletions (InDel) variants (Figures 7, 8). It was observed that for *pfhrp2*, the variations were mostly as a result of missense variants whilst for *pfhrp3*, they were as a result of frameshift variants as shown in Figures 7, 8. For *pfhrp2*, only 6 isolates had the wildtype amino acid sequence of 282 amino acids in the 1,124 isolates. Hundred (100) isolates had about 40 amino acids being the majority. A similar observation was made for *pfhrp3* with a wildtype amino acid sequence of 252 seen in only 3 isolates. About 219 isolates had 223 amino acids being the majority out of the 1,293. The extent of deletions and insertions in the exon 2 of these genes in the isolates are diverse. The mechanisms underlining these variations are shown in Figures 7, 8.

### Haplotype diversity analysis

The haplotype diversity metric was used to measure the genetic diversity at exon 2 of the *pfhrp2* and *pfhrp3*. Haplotype diversity measures the probability that two randomly chosen parasite gene sequences are different. We aimed to determine whether haplotype diversity varied among the three time points and transmission zones of our study. Our analysis showed high haplotype diversity for all zones across all time points was between 0.872 and 1 for both genes (Table 4).

**Table 4:** Haplotype diversity of *pfhrp2* and *pfhrp3* genes.

**Gene**	**Zone**	**Time point**	**Number of samples**	**Number of haplotypes**	**Haplotype diversity**
*pfhrp2*	Coastal	2015_2016	103	99	0.999
		2017_2018	54	54	1
		2019_2020	48	48	1
	Forrest	2015_2016	192	187	0.9992
		2017_2018	280	210	0.9482
		2019_2020	272	243	0.9967
	Savannah	2015_2016	49	49	1
		2017_2018	33	33	1
		2019_2020	108	98	0.997
*pfhrp3*	Coastal	2015_2016	93	91	0.999
		2017_2018	71	62	0.985
		2019_2020	65	52	0.956
	Forrest	2015_2016	164	152	0.9963
		2017_2018	362	295	0.9737
		2019_2020	195	121	0.872
	Savannah	2015_2016	94	80	0.982
		2017_2018	169	143	0.9753
		2019_2020	80	55	0.919

There were no observed differences between the estimated haplotype diversities of the two genes for the isolates, either by time periods or transmission zones.

### Variation in the frequency of repeats in the *Pfhrp2* for Baker model types

**Figure 9:**
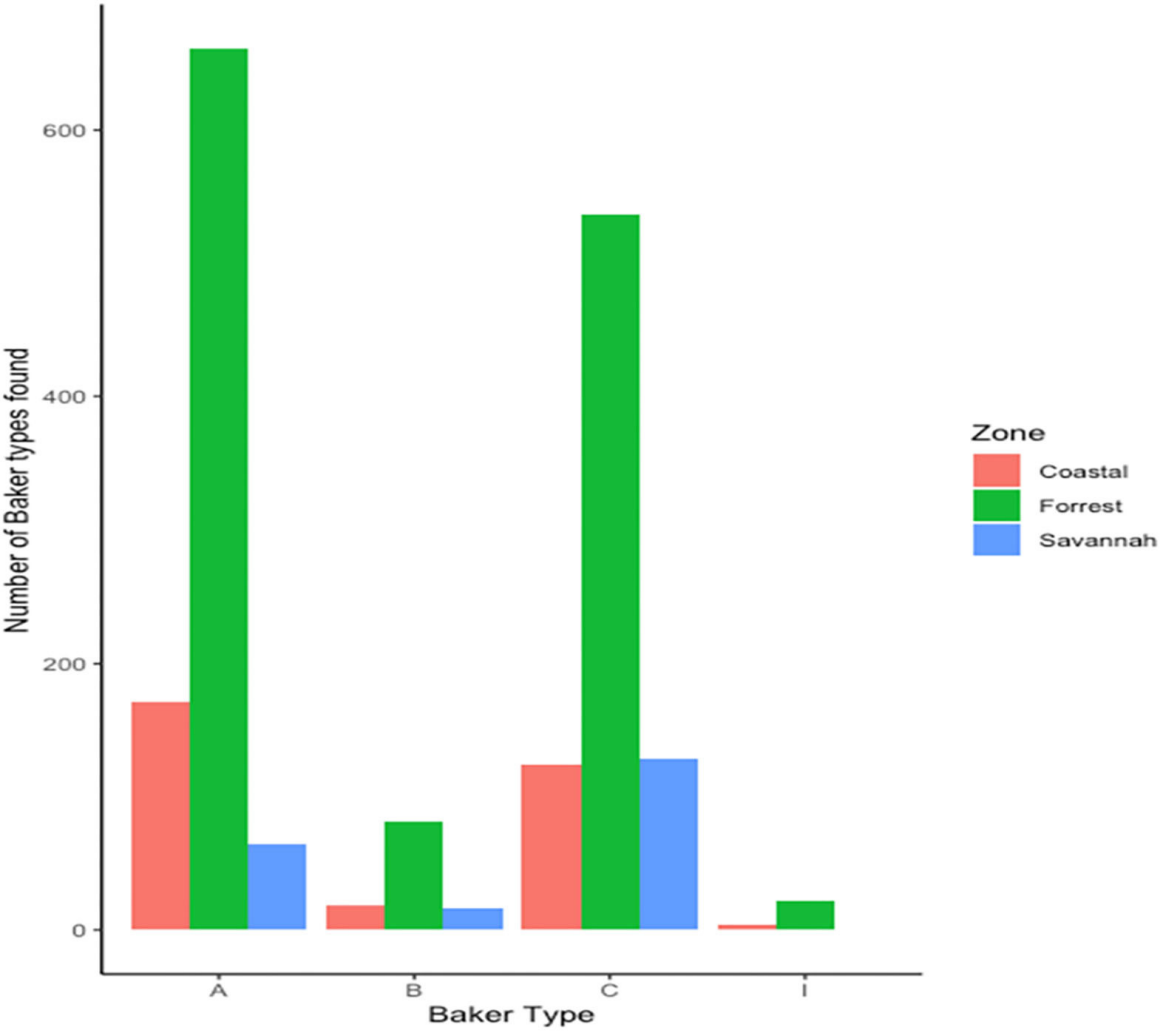
Distribution of Baker model types across the three ecological zones.

We found 17 of the known Baker amino acid repeats (14) in both *pfhrp2* and *pfhrp3* exon 2 in samples analyzed. Samples contained varied repeat number of these 17 amino acid repeat types as already shown in Table 2. Groups are defined as A, B, I and C if their Baker repeat (type 2 × type 7) was >100, 50–100, 44–49, and <43, respectively which includes all variants of the two types. The frequencies of occurrence of Baker model types for the three ecological zones are shown in Table 5. In all, the Baker types were dominant in the forest zone (Figure 9).

**Table 5:** Distribution of Baker types in *pfhrp2* exon 2 across the three transmission zones.

**Zone**	**Bakers type**				**Total**
	**A (>100)**	**B (50–100)**	**I (44–49)**	**C (<43)**	
Coastal	171	18	4	125	318
Forrest	661	81	22	537	1,301
Savannah	64	16	0	129	209
Total	896	115	26	791	1,828

Single nucleotide polymorphisms (SNP) variants of each type (subvariants) were included in this analysis.

### Association between Baker's model types from the ecological zones and RDT sensitivity

Both *pfhrp2* and *pfhrp3* genes are located in the subtelomeric regions of chromosomes 8 and 13 (12) respectively, therefore the hypothesis that diversity in these genes is driven by sexual recombination rates in the mosquito vector which is in turn a function of local transmission rates and multiplicity of infections (MOI) was tested. The hypothesis was tested by using the three transmission zones as proxies of sexual recombination rate with guinea savannah greater than forest and forest greater than coastal savannah because samples have been already controlled with MOI greater than one. A 2 × 2 contingency table was generated with the response variables as predicted RDT sensitivity using Baker's model (14) and the predictor variables as the three transmission zones (Table 6). Baker's model had earlier shown that the product of type 2 and type 7 repeat numbers correlates with PFHRP2-RDT sensitivity detection (14). With a chi squared value of 33.131, with 2 degrees of freedom and a *p*-value of 6.392 × 10.08, it shows a statistically significant association between transmission zones and predicted RDT sensitivity. To advance this further, the strength of the association between each transmission zone (using coastal zone as our reference level) and predicted RDT sensitivity using a logistic regression model was determined. A statistically significant association was observed between guinea savannah zone and odds of RDT sensitivity (odds ratio = 0.40, 90% confidence interval = 0.28–0.57, *p*-value < 0.001).

**Table 6:** Chi-square test conducted on 2 × 2 contingency table generated by collapsing groups A, B and I of Table 5 into sensitive column and group C remaining as non-sensitive.

**Ecological zone**	**Predicted RDT sensitivity by Baker's model**	
	**Sensitive**	**Not sensitive**
Coastal savannah	193	125
Forest	764	537
Guinea savannah	80	129

Null hypothesis was that there was no association between transmission zone and predicted RDT sensitivity by Baker's model. Level of significance, alpha, was set at 0.05. χ2 = 33.131, df = 2, p-value = 6.392e-08.

## Discussion

The WHO has recommended the use of RDTs in the absence of microscopy for diagnosing malaria in disease endemic countries. PFHRP2-based RDTs play a critical role in the diagnosis of *P. falciparum* malaria throughout sub-Saharan Africa. However, reports of *P. falciparum* isolates lacking the *pfhrp2* gene encoding the PFHRP2 protein as well as the existence of isolates with enormous *pfhrp2* diversity pose a challenge to the continued use of the RDTs (16, 32). Due to the many advantages of PFHRP2-based RDTs, a high prevalence of the gene deletions could have a far-reaching effect on *P. falciparum* malaria case management and consequently disease elimination and eradication. This population genetics study thus investigated the diversity of the *pfhrp2* and *pfhrp3* genes in *P. falciparum* clinical isolates from Ghana from 2015 to 2020. The findings showed high prevalence of deletions with increasing trends across the years from 2015 to 2020, an enormous diversity of the amino acid type variants of *pfhrp2* and *pfhrp3* and its associated reduced RDT sensitivity for the detection of the malaria parasite population in Ghana.

This study observed deletions in 30.7 and 17.2% of the isolates for *pfhrp2* and *pfhrp3* genes respectively. Comparatively similar range of prevalence of *pfhrp2* deletions have been observed in studies conducted in Ghana (36%), Guyana (41%), Surinam (14%), Rwanda (23%) and Peru (41%) (16, 18, 22, 33). The differences in the prevalence could be due to sample size of the studies and the malaria situation in the study sites. In sub-Saharan Africa, specifically Ghana, Rwanda, and the Democratic Republic of the Congo, there are substantial proportions of *pfhrp2* deleted isolates and it is quite a problem since malaria diagnosis is predominately reliant on PFHRP2-RDTs. The possibility of false negative results with such RDTs cannot be overemphasized especially in comparison with other studies that observed none of such gene deletions in their parasite populations (12, 14, 29). Comparing sample sizes of work done so far on the deletions of *pfhrp2* and *pfhrp3* deletions, this population genetic study used the largest number of samples and a leverage of three time points to detect the spread of the mutation over time. It is also significant to mention that this study's analysis of a large sample size as compared to limited numbers seen in other studies has highlighted the extent of major deletions and insertions in the exon 2 of the genes in isolates which were *pfhrp2* and *pfhrp3* positive. Studies conducted in Ghana such as by Addai-Mensah and others observed no deletions in the middle belt of Ghana (Volta Region) with a sample size of 50, whereas Amoah and others observed deletions in Ghanaian isolates in coastal part of Ghana (24, 34). The observation made by Amoah and others is corroborated by our study that there are geographical or regional differences in the prevalence of the deletions. With regards to the time points incorporated in the study analysis, an increasing trend in the deletions were observed which was lacking in previous studies. The time points analysis portrays the selection and spread of such mutations in the parasite population and could be used for modeling for prediction analysis. Another aspect that is important to mention is the differences in ecological zones because of variability in transmission dynamics and consequent genetic recombination rates contributing to the diversity observed within the parasite population.

The *pfhrp2* gene had 17 of the known repeat types as described by Baker et al. (12) in this study. Interestingly, repeat types 19 (AHHAA) and 21 (AHHAHHATY) observed in this study is in accordance with the types seen by Baker et al. however, they have not been reported in Africa and some other countries (7, 24, 29). There was an absence of the repeat type 9 (AAY) in this study but this type has been seen in isolates from African countries and elsewhere (21, 29, 35–37). The diversity in the occurrence of these types in the different regions of the world could be as a result of random mutation and local selection and spread of *pfhrp2* mutated strains (12, 38). The *pfhrp3* gene of the Ghanaian isolates had 17 known repeat types and surprisingly, repeat types 9 and 19 which are hardly observed in *P. falciparum* isolates from around the world were seen in this study (7, 24, 29). Comparatively, this study observed more *pfhrp3* repeat types than other studies which could also be due to the large sample size analyzed. Ten repeat types 1, 2, 3, 4, 5, 6, 7, 8, 10, 11,12,13,14, 15, and 19, were shared between the *pfhrp2* and *pfhrp3* although they are located on two different chromosomes. The relevance of this observation is the cumulative effect due to the two genes to enhance sensitivity of the RDTs.

The genetic diversity of *pfhrp2* in isolates was more pronounced as compared to that of *pfhrp3* which is consistent with reports from Madagascar, Senegal, Central America, Ethiopia and Kenya (13, 15, 21, 29, 35). The high haplotype diversity seen in the isolates could be as a result of the location of the *pfhrp2* and *pfhrp3* genes in the subtelomeric regions and are highly susceptible to changes due to high recombination rates (12–14, 39). In addition, the variations could be a result of random mutation, geographical and transmission setting differences, level of immunity of study population, frequency of exposure to drug as well as possibly the methods used for the investigation (40–43). However, the data did not implicate an association between haplotype diversity and transmission zones or with the time points. Numerous variants of the repeat types 1, 2, 3, 4, 5, 6, 7, 8, 10, 12, 13, 14, and 15 were observed for the *pfhrp2* in Ghanaian isolates. These variants were as a result of insertions and deletions and others as a result of frame shift as well as major substitution of ≥1 amino acid. It was interesting to observe that an amino acid reference size of *pfhrp2* exon 2 is 282 amino acids and there were isolates with only 20 amino acids for exon 2 (major deletion) being the lowest and others with 325 (major insertion) as the highest number of amino acids. A similar observation was made for *pfhrp3*, with an exon 2 reference size of 252 amino acids, however isolates had between 20 and 302 amino acids. This portrays the variability of the genes in the isolates and this may have dire consequences for RDT sensitivity due to differences protein conformations.

The dominant amino acid variants observed in the *pfhrp2* gene, the types, AHHAHHVAH, AHHAHHAHD, APHAHHAHH, APHAHHAPH, APHAPHAPH, APH, AHHAHHASH, APHAPH and AHHAPY have not been reported elsewhere and are therefore novel variants in the parasites in Ghana. The observation of the type 2, AHHAHHAPD, AHHAHHAAH, AHHAHHADD and the type 7, AHHADD and AHHAAH variants in the study however is not surprising as these variants were also observed in a study conducted in the Volta region (forest zone) in the country (24) though the sample size for that study was comparatively small. Similar variants of the two types have been reported in studies conducted in other African countries and India (29, 35, 44). For the *pfhrp3* gene, the variants AHHATN, AHHNA, SHDDH and SHHDH are also novel and only observed in Ghanaian isolates. Some of the observed prominent variants such as AHHAAH, PHHDG, SHHDG, PHHDD, AHHAPH have also been reported in Kenya and Ethiopia (29, 35) indicating that these variants may be circulating amongst the African *P. falciparum* population.

The genetic diversity of *pfhrp2* and its effect on RDT performance is crucial for sensitivity and accurate test results in malaria diagnosis. The two most common amino acid repeat sequences observed in the study for the *pfhrp2* were the type 2 (AHHAHHAAD) and type 7 (AHHAAD). The abundance of these repeat sequences has been linked to an increase in RDT sensitivity especially with parasite densities of ≤ 250 parasites/μl (14, 29). Since both repeats have been used as epitopes for monoclonal antibodies for the RDTs, it implies that a higher epitope frequency within a sequence may result in greater sensitivity (14, 21, 45). However, some studies have contradicted this assertion and have associated types 2 and 7 with RDT false negativity and reduced detection below a certain threshold (19, 21). It is quite interesting to observe the enormous variants of these two types in the isolates and this may have a negative impact of the sensitivity due to change in protein conformation and resultant specificity of antigen binding affinity to monoclonal antibodies on the RDT cassette. Monoclonal antibodies specific for PFHRP2 are reported to be able to detect PFHRP3 and this cross-reactivity is influenced by the presence of amino acid types 1, 2, 4, and 7 repeats (14).

Using the Baker's regression model, the finding suggests that the odds of testing positive using PFHRP2 RDT kits in the guinea savannah zone is more than halved compared to the coastal savannah and forest zones. It further suggests that the relatively higher transmission intensity of the guinea savannah zone, and its corollary effect of increased sexual recombination, is a driver of diversity at the *pfhrp2* subtelomeric locus and may have led to the evolution of *pfhrp2* variants with reduced sensitivity to PFHRP2 RDT monoclonal antibodies. A limitation to this analysis pipeline that may impact the conclusion is our use of the Baker's regression model to assign RDT sensitivity classes, as the model was largely built from data derived from laboratory parasite strains using RDTs in 2005.

## Conclusion

The findings from this population genetics study of the *pfhrp2* and *pfhrp3* genes of Ghanaian isolates has implications for RDT sensitivity in malaria diagnosis in the country. The high prevalence of the deletions of the genes and the increasing trend in the prevalence of these deletions over time is indicative of reduced sensitivity and failure of these RDT overtime. Generally, the findings from using this large sample size with different timepoints and ecologies has highlighted the increasing reduction of RDT sensitivity and its consequent false negatives in malaria diagnosis and calls for continuous surveillance as part of the ongoing TES in malaria endemic countries.

## Data availability statement

All data generated or analyzed during this study are included in this published article. Raw genetic data on some of the sequences have been deposited at the NCBI with accession numbers from OP329724 to OP329735 and from OP413719 to OP413737.

## Ethics statement

The studies involving human participants were reviewed and approved by Noguchi Memorial Institute for Medical Research Institutional Review Board (IRB CPN 015/19-20) and the US Naval Medical Research Center (NMRC) (NAMRU3.2019.0002) in compliance with all applicable federal regulations governing the protection of human subjects. Written informed consent to participate in this study was provided by the participants' legal guardian/next of kin.

## Author contributions

ND-Q, DW, CW, NQ, BA, HQ, and TS conceived and designed the study. PO-A, SB, TA, KT, NE, and SM did the laboratory analysis of samples to generate molecular data as well as the sequence analysis. ND-Q drafted the manuscript. All authors read, reviewed and approved the final manuscript.

## Funding

This work, which includes the collection of the samples used in this study, was funded by the Global Emerging Infections Surveillance and Response Section (GEIS), a division of the US Armed Forces Health Surveillance Center (AFHSC) (ProMIS ID P0142_19_N3) and the Global Fund to fight Aids, Tuberculosis and Malaria (GFATM)/National Malaria Control Program, Ghana.

## Conflict of interest

The authors declare that the research was conducted in the absence of any commercial or financial relationships that could be construed as a potential conflict of interest.

## Publisher's note

All claims expressed in this article are solely those of the authors and do not necessarily represent those of their affiliated organizations, or those of the publisher, the editors and the reviewers. Any product that may be evaluated in this article, or claim that may be made by its manufacturer, is not guaranteed or endorsed by the publisher.
