# Supplementary material for: Genetic deletions and high diversity of Plasmodium falciparum histidine-rich proteins 2 and 3 genes in parasite populations in Ghana
Source: Front Epidemiol. 2022 Oct 14;2:1011938. doi: 10.3389/fepid.2022.1011938 (PMC10911008; doi:10.3389/fepid.2022.1011938)
Supplement: Supplementary file 1 [file Table_1.DOCX]

|  | | | |
| --- | --- | --- | --- |
| Supplementary Table 1: *Pfhrp2* variants observed in all samples  The normal amino acid repeat types, variants of the types and the number of samples they were observed in are in brackets. Those in bold are the variants occurring in samples from 10 and above. | | | |
|  |  |  |  |
| Repeat Type | Repeat Sequence | Variant | Total no. of samples with variants |
| 1 | AHHAHHVAD | AHHADHVAD | 2 |
|  |  | **AHHAHHVAH** | **15** |
|  |  | AHHAHHVAN | 2 |
|  |  | AHHAHHVPD | 7 |
|  |  | AHHAHHVDD | 1 |
|  |  | AHHAHHVLD | 1 |
|  |  | AHHAHHVPH | 3 |
|  |  | AHHAHHVTD | 3 |
|  |  | AHHAHHVTN | 1 |
|  |  | AHHAHHVSD | 1 |
|  |  | AHHAHHVYH | 1 |
|  |  | AHHAHHYYA | 1 |
|  |  | AHHAHNVAD | 2 |
|  |  | AHHAHYVAD | 1 |
|  |  | AHHDHHVAD | 1 |
|  |  | AHHGHHVSD | 1 |
|  |  | AHLAHHVPD | 1 |
|  |  | AHQAHHVAD | 3 |
|  |  | APHAHHVAD | 1 |
|  |  | AQHAHHVAD | 1 |
|  |  | AQHADHVAD | 1 |
|  |  | CHHAHHVAD | 4 |
|  |  | SHHAHHVAD | 2 |
|  |  | DHHAHHVAD | 1 |
|  |  | THHAHHVAD | 1 |
| 2 | AHHAHHAAD | AAHAHHAAH | 1 |
|  |  | AHAHHAHD | 1 |
|  |  | AHDAHHAHD | 1 |
|  |  | AHDAHHAHH | 2 |
|  |  | AHHAAHAHH | 1 |
|  |  | AHHAAHAPD | 1 |
|  |  | AHHAAPHHEAATH | 1 |
|  |  | AHHAAPHHATD | 1 |
|  |  | AHHAAPAHAAD | 1 |
|  |  | AHHADHAAH | 1 |
|  |  | AHHADHADD | 1 |
|  |  | AHHADHAPD | 1 |
|  |  | AHHADHAPH | 1 |
|  |  | AHHAHDADD | 1 |
|  |  | AHHAHDAHD | 1 |
|  |  | AHHAHDAHH | 1 |
|  |  | **AHHAHHAAH** | **110** |
|  |  | **AHHAHHADD** | **25** |
|  |  | AHHAHHAAA | 2 |
|  |  | AHHAHHADH | 7 |
|  |  | **AHHAHHAHD** | **55** |
|  |  | AHHAHHADH | 3 |
|  |  | AHHAHHADY | 2 |
|  |  | **AHHAHHAHH** | **214** |
|  |  | AHHAHHALD | 1 |
|  |  | AHHAHHAPA | 1 |
|  |  | AHHAHHAND | 1 |
|  |  | **AHHAHHAPD** | **276** |
|  |  | **AHHAHHAPH** | **402** |
|  |  | AHHAHHAPN | 4 |
|  |  | AHHAHHAPP | 1 |
|  |  | AHHAHHAYH | 5 |
|  |  | AHHAHHAYD | 3 |
|  |  | AHHAHHGAD | 1 |
|  |  | AHHAHHETD | 1 |
|  |  | AHHAHHTPH | 1 |
|  |  | AHHAHLAAD | 1 |
|  |  | AHHAHNAAD | 3 |
|  |  | AHHAHVAAH | 1 |
|  |  | AHHAHYAPD | 2 |
|  |  | AHHAHQAAD | 3 |
|  |  | AHHAHYAAD | 2 |
|  |  | AHHAKHAAD | 1 |
|  |  | AHHAHYAPH | 3 |
|  |  | AHHANHAAD | 1 |
|  |  | AHHANHATD | 1 |
|  |  | AHHAPDAHH | 4 |
|  |  | AHHAPDAPD | 1 |
|  |  | AHHAPDAPH | 1 |
|  |  | AHHAPHAAH | 1 |
|  |  | AHHAPHADH | 1 |
|  |  | AHHAPHAHD | 2 |
|  |  | **AHHAPHAHH** | **86** |
|  |  | **AHHAPHAPH** | **26** |
|  |  | AHHAPHAPD | 2 |
|  |  | AHHAPHAPP | 1 |
|  |  | AHHAPHASH | 1 |
|  |  | AHHAPYAPH | 1 |
|  |  | AHHAQHAAH | 1 |
|  |  | AHHAQHASD | 1 |
|  |  | AHHAQHAAD | 1 |
|  |  | AHHAYHAAD | 3 |
|  |  | AHHAYHAPD | 1 |
|  |  | AHHDHHAAD | 1 |
|  |  | AHHLHHAAD | 1 |
|  |  | AHHTHHAAD | 1 |
|  |  | AHHTPHAPH | 1 |
|  |  | AHHVHNAAD | 1 |
|  |  | AHHTHHAAH | 1 |
|  |  | AHNAHHAAD | 2 |
|  |  | AHNAHHAPD | 1 |
|  |  | AHPAHHAAD | 1 |
|  |  | AHQAHHAAD | 4 |
|  |  | AHPAPHAPH | 1 |
|  |  | AHQAHHAAD | 3 |
|  |  | AHQDHHVAD | 2 |
|  |  | AHYAHHAAH | 1 |
|  |  | AHYAHHAAD | 1 |
|  |  | AHYAHYAPH | 1 |
|  |  | ANHAHHAAD | 1 |
|  |  | APHAHHAAD | 7 |
|  |  | APHAHHAAH | 7 |
|  |  | **APHAHHAHH** | **13** |
|  |  | APHAHHAPD | 2 |
|  |  | **APHAHHAPH** | **37** |
|  |  | APHAPHAHH | 2 |
|  |  | APHAPHAAH | 1 |
|  |  | **APHAPHAPH** | **10** |
|  |  | AQHAHHAAD | 1 |
|  |  | DHHAHHAAD | 2 |
|  |  | AYHAHHAAD | 2 |
|  |  | THHAHHAAD | 1 |
|  |  | THDAHHADD | 1 |
|  |  | THHAHHAAD | 1 |
|  |  | THQAHHAAD | 1 |
|  |  | YHHAHHAAD | 3 |
|  |  | YYHAHHAAD | 1 |
| 3 | AHHAHHAAY | AHHAHHAHY | 2 |
|  |  | **AHHAHHAPY** | **57** |
|  |  | AHHAPHAAY | 1 |
|  |  | AHHAQHAAY | 1 |
|  |  | AHQAHHAAY | 1 |
|  |  | APHAHHAAY | 1 |
|  |  | DHHAHHAAY | 1 |
|  |  | THHAHHAAY | 1 |
| 4 | AHH | ADD | 1 |
|  |  | ADH | 1 |
|  |  | **APH** | **11** |
| 5 | AHHAHHASD | AHHAHDASH | 1 |
|  |  | AHHAHDASH | 1 |
|  |  | **AHHAHHASH** | **51** |
|  |  | AHHAHHASP | 1 |
|  |  | AHHAHHASY | 1 |
|  |  | AHHDRHASD | 1 |
| 6 | AHHATD | **AHHATH** | **22** |
|  |  | THHATD | 1 |
| 7 | AHHAAD | AAHAHH | 2 |
|  |  | ADDADH | 1 |
|  |  | ADDAHD | 1 |
|  |  | ADHADD | 2 |
|  |  | ADHADH | 1 |
|  |  | ADHAHH | 1 |
|  |  | ADHAHD | 1 |
|  |  | ADHAHH | 3 |
|  |  | ADHAPH | 1 |
|  |  | AHDADD | 2 |
|  |  | AHDAAH | 1 |
|  |  | AHDADH | 1 |
|  |  | AHDAHD | 2 |
|  |  | AHDAHH | 4 |
|  |  | AHHAAA | 1 |
|  |  | **AHHAAH** | **44** |
|  |  | **AHHADD** | **26** |
|  |  | **AHHADH** | **12** |
|  |  | **AHHAHD** | **56** |
|  |  | AHHAND | 1 |
|  |  | **AHHAPD** | **121** |
|  |  | AHHANH | 1 |
|  |  | **AHHAPH** | **246** |
|  |  | AHHAPN | 3 |
|  |  | AHHAYH | 4 |
|  |  | AHHAYL | 1 |
|  |  | AHHDTD | 1 |
|  |  | AHHVAD | 1 |
|  |  | AHPAHH | 1 |
|  |  | APDAPD | 1 |
|  |  | APHAAD | 1 |
|  |  | APHAAH | 1 |
|  |  | **APHAHH** | **44** |
|  |  | APHADH | 1 |
|  |  | **APHAPH** | **10** |
|  |  | AYHAHH | 1 |
|  |  | DYHAHH | 1 |
| 8 | AHHAAY | **AHHAPY** | **22** |
|  |  | AHHATY | 1 |
| 10 | AHHAAAHHATD | ADHAAAHHATD | 2 |
|  |  | AHHAAADHAAD | 4 |
|  |  | AHHAAADHDAD | 1 |
|  |  | AHHAAADHATD | 1 |
|  |  | AHHAAAHHAAD | 4 |
|  |  | AHHAAAHHAAD | 1 |
|  |  | AHHAAAHHAAD | 1 |
|  |  | AHHAAAHHAND | 1 |
|  |  | AHHAAAHHATG | 1 |
|  |  | AHHAAAHHAND | 2 |
|  |  | AHHAAAHHEAD | 1 |
|  |  | AHHAAAHHETD | 1 |
|  |  | AHHAADDDDAD | 1 |
|  |  | AHHAADAHHAA | 1 |
|  |  | AHHAADDHDAD | 2 |
|  |  | AHHAADHPADD | 1 |
|  |  | AHHAAEDHAAD | 1 |
|  |  | AHHAAEDHDTD | 1 |
|  |  | AHHAAEHHATD | 1 |
|  |  | AHHAAHAHATD | 2 |
|  |  | AHHADAHHATD | 2 |
|  |  | AHHADHHASD | 1 |
|  |  | AHHAHAHHATD | 1 |
|  |  | AHHAHDAHAHD | 1 |
|  |  | AHHAHDAHATD | 1 |
|  |  | AHHAHDDHATD | 1 |
|  |  | AHHAHDHHATD | 1 |
|  |  | AHHAPDAHATD | 1 |
|  |  | AHHAPDDHATD | 1 |
|  |  | AHHAPHAHHAD | 1 |
|  |  | AHHAPPHHATD | 1 |
|  |  | AHHATAHHATD | 2 |
|  |  | AHHVAAHHATD | 1 |
|  |  | APHAAAHHATD | 1 |
|  |  | DHHAAAHHATD | 1 |
|  |  | QHATDAHHATD | 1 |
| 12 | AHHAAAHHEAATH | ADHEAAHDEAATH | 1 |
|  |  | AHHAAADHEAAAH | 1 |
|  |  | AHHAAADHEAATH | 1 |
|  |  | AHHAAAHDEAATH | 1 |
|  |  | AHHAAAHHEAADH | 1 |
|  |  | AHHAAAHHATY | 1 |
|  |  | AHHAAAHHDTD | 1 |
|  |  | AHHAAAHHEAAEH | 1 |
|  |  | AHHAAAHHEAAPH | 1 |
|  |  | AHHAAAHHEFATH | 1 |
|  |  | AHHAAAHHEDATH | 1 |
|  |  | AHHAAAHHEVATH | 1 |
|  |  | AHHAAAPHEAATH | 1 |
|  |  | AHHADAHHEAAAH | 2 |
|  |  | AHHADAHHEAATH | 1 |
|  |  | AHHADDHHEAD | 1 |
|  |  | AHHAVAHHEAATH | 1 |
|  |  | AHHEAAHDEAATH | 1 |
| 13 | AHHASD | AHHASH | 7 |
| 14 | AHHAHHATD | AHHAHHATH | 3 |
|  |  | AHHAHHATY | 2 |
|  |  | APHAHHATH | 2 |
| 15 | AHHAAN | AHHASN | 1 |

**Supplementary Table 2: *pfhrp3* variants observed in all samples**

The normal amino acid repeat types, variants of the types and the number of samples they were observed in are in brackets. Those in bold are the variants occurring in samples from 10 and above.

| Repeat Type | Repeat Sequence | Variant | Total no. of samples with variants |
| --- | --- | --- | --- |
| 1 | AHHAHHVAD | AHHAHHAGD | 3 |
|  |  | AHHAHHVAE | 1 |
|  |  | AHHAHHVAH | 6 |
|  |  | AHHAHHVAN | 1 |
|  |  | AHHAHHVAY | 1 |
|  |  | AHHAHHVDH | 1 |
|  |  | AHHAHHVHH | 3 |
|  |  | AHHAHHVPD | 7 |
|  |  | AHHAHHVPH | 1 |
|  |  | AHHAHHVTD | 1 |
|  |  | AHHAHNASL | 1 |
|  |  | AHHAHQVAD | 1 |
|  |  | AHHAHRVAD | 2 |
|  |  | AHHALHVAD | 1 |
|  |  | AHHANHVAD | 1 |
|  |  | AHHAPHVPD | 1 |
|  |  | AYHAHHVAD | 1 |
|  |  | CHHAHHVAD | 4 |
|  |  | CHHCHHVAD | 1 |
| 3 | AHHAHHAAY | AHHAHHASY | 1 |
| 4 | AHH | AAH | 1 |
|  |  | AAN | 1 |
|  |  | AHD | 1 |
|  |  | APN | 1 |
|  |  | AQH | 1 |
|  |  | AQN | 1 |
| 7 | AHHAAD | AHHADD | 1 |
|  |  | AHHAFD | 2 |
|  |  | AHHAGD | 1 |
|  |  | AHHAPD | 4 |
| 8 | AHHAAY | AHHAHY | 1 |
|  |  | AHHASY | 2 |
|  |  | AHHATY | 1 |
| 15 | AHHAHHAAN | AHHAHHAAH | 1 |
|  |  | AHHAHHAAM | 1 |
|  |  | AHHAHHAAP | 1 |
|  |  | AHHAHHAPH | 3 |
|  |  | AHHAHHAPN | 5 |
|  |  | AHHAHHASH | 1 |
|  |  | AHHAHHASN | 1 |
|  |  | APHAPHAPH | 1 |
|  |  | APNAHHAAN | 1 |
|  |  | LHHAHHAAN | 1 |
| 16 | AHHAAN | AHHAAE | 1 |
|  |  | **AHHAAH** | **208** |
|  |  | AHHAAI | 1 |
|  |  | AHHAAK | 6 |
|  |  | AHHAAM | 8 |
|  |  | AHHAAR | 1 |
|  |  | AHHAAT | 2 |
|  |  | AHHADH | 1 |
|  |  | AHHADN | 1 |
|  |  | AHHAFH | 2 |
|  |  | AHHAHN | 1 |
|  |  | AHHANH | 1 |
|  |  | AHHANN | 1 |
|  |  | AHHAPA | 1 |
|  |  | **AHHAPH** | **149** |
|  |  | AHHAPK | 1 |
|  |  | **AHHAPN** | **51** |
|  |  | AHHASA | 1 |
|  |  | AHHAPY | 1 |
|  |  | **AHHASH** | **77** |
|  |  | AHHASK | 1 |
|  |  | AHHATH | 1 |
|  |  | **AHHATN** | **38** |
|  |  | AHHAYH | 1 |
|  |  | AHHDSN | 1 |
|  |  | AHHGSY | 1 |
|  |  | AHHTAN | 1 |
|  |  | AHHVAN | 1 |
|  |  | AHYASL | 1 |
|  |  | APHAAN | 2 |
|  |  | APHAPH | 3 |
|  |  | ANHAAN | 3 |
|  |  | APNAAN | 1 |
|  |  | DHHAAN | 1 |
|  |  | EHHAAN | 1 |
|  |  | HHHAAN | 1 |
|  |  | PHHAAN | 1 |
|  |  | THHAAN | 1 |
| 17 | AHHDG | ADHDE | 1 |
|  |  | AHANA | 1 |
|  |  | AHHAN | 5 |
|  |  | AHHDA | 8 |
|  |  | **AHHDE** | **63** |
|  |  | **AHHDH** | **11** |
|  |  | AHHDQ | 2 |
|  |  | **AHHNA** | **244** |
|  |  | AHHNE | 1 |
|  |  | AHHVA | 1 |
|  |  | APHDG | 1 |
|  |  | ATHDE | 1 |
|  |  | DHHDG | 1 |
|  |  | PGHDG | 2 |
|  |  | PHHDE | 5 |
|  |  | **PHHDG** | **33** |
|  |  | **SHHDG** | **22** |
|  |  | THHDD | 1 |
|  |  | THHDG | 1 |
|  |  | TTHDG | 1 |
| 18 | AHHDD | ADHDD | 3 |
|  |  | AHHAD | 1 |
|  |  | AHHDY | 1 |
|  |  | AHHED | 1 |
|  |  | AHHHD | 2 |
|  |  | AHHND | 5 |
|  |  | APHDD | 1 |
|  |  | DHHDD | 2 |
|  |  | EHHDE | 2 |
|  |  | EHHDD | 6 |
|  |  | HHHDD | 2 |
|  |  | HHHDG | 1 |
|  |  | **PHHDD** | **19** |
| 20 | SHHDD | SDHDD | 1 |
|  |  | SHDDD | 3 |
|  |  | **SHDDH** | **14** |
|  |  | SHDDN | 1 |
|  |  | **SHHDH** | **83** |
|  |  | SHHDY | 1 |
