# Supplementary material for: Genetic deletions and high diversity of Plasmodium falciparum histidine-rich proteins 2 and 3 genes in parasite populations in Ghana
Source: Front Epidemiol. 2022 Oct 14;2:1011938. doi: 10.3389/fepid.2022.1011938 (PMC10911008; doi:10.3389/fepid.2022.1011938)
Supplement: Supplementary file 2 [file Table_2.DOCX]

**Supplementary Table 3**

Primer sequences used for *pfhrp2* and *pfhrp3* gene amplifications and the cycling parameters for both the primary and secondary PCRs.

| **Primer name** | **Sequence** |
| --- | --- |
| Pfhrp2-F1 | CAAAAGGACTTAATTTAAATAAGAG |
| Pfhrp2-R1 | ATTATTACACGAAACTCAAGCAC |
| Pfhrp2- F2 | ATTATTACACGAAACTCAAGCAC |
| Pfhrp3-F1 | AATGCAAAAGGACTTAATTC |
| Pfhrp3-R1 | TGGTGTAAGTGATGCGTAGT |
| Pfhrp3-F2 | AAATAAGAGATTATTACACGAAAG |
|  |  |
| **Cycling conditions** |  |
| Primary PCR | 94°C for 10mins  94°C for 50s, 55°C for 50s, 72°C for 1min (x35)  72 °C for 10 mins |
| Secondary PCR | 94°C for 10mins  94°C for 50s, 55^o^C for 50s, 72^o^C for 1min (x37)  72°C for 10mins |
